# Facilitators of medication adherence in patients with hypertension: a qualitative study

**DOI:** 10.3389/fpubh.2024.1372698

**Published:** 2024-11-19

**Authors:** Zahra Ghaderi Nasab, Hamid Sharifi, Parvin Mangolian Shahrbabaki

**Affiliations:** ^1^MSc Student, Department of Biostatistics and Epidemiology, School of Public Health, Kerman University of Medical Sciences, Kerman, Iran; ^2^HIV/STI Surveillance Research Center, and WHO Collaborating Center for HIV Surveillance, Institute for Futures Studies in Health, Kerman University of Medical Sciences, Kerman, Iran; ^3^Institute for Global Health Sciences, University of California, San Francisco, San Francisco, CA, United States; ^4^Student Research Committee, Kerman University of Medical Sciences, Kerman, Iran

**Keywords:** hypertension, medication adherence, facilitator, content analysis, qualitative study

## Abstract

Hypertension has been identified as the most important risk factor for disability and premature death worldwide. This study aimed to explain the factors that facilitate medication adherence from the perspective of patients with hypertension in southeastern Iran. This qualitative study used conventional content analysis and purposive sampling methods to achieve data saturation, involving 25 participants. Facilitating factors were categorized into two main categories: individual and family factors and organizational support. Individual factors included motivational fears, disease acceptance, healthy lifestyle, disease monitoring, and follow-up. Family factors included family support, while organizational support included governmental support to provide inexpensive medicines, comprehensive healthcare team support, health insurance access, and media training. These factors were identified as essential facilitators that significantly influence appropriate adherence to hypertensive drugs. This study revealed that various factors affected medication adherence in these patients, and efforts should be made to improve hypertension treatment and increase medication adherence. Measures taken to control hypertension are cost-effective to reduce the burden associated with this disease. The findings of this study can be valuable in formulating policies for the treatment and care of hypertension.

## 1 Introduction

Hypertension is the primary and most common risk factor for cardiovascular disease, stroke, and kidney disease. It is recognized as the most important risk factor for disability and premature death in the world (1). Hypertension is responsible for at least 45% of cardiovascular disease-related deaths and 51% of stroke-related deaths (2). It is estimated that 10% of total healthcare costs are directly related to hypertension and its complications (3). One in three adults worldwide suffers from hypertension, with this rate increasing with age (2). Prevalence of hypertension 25% In Iranian adults, it is estimated that this number is growing (4). The increasing prevalence of this disease and its related complications can be attributed mainly to population growth and lifestyle changes (5). However, it should be noted that adopting a healthy lifestyle, including a balanced diet, abstaining from smoking and excessive alcohol consumption, and engaging in regular physical activity, can reduce systolic blood pressure by 3.5 mm Hg and lower the risk of cardiovascular disease by approximately 30% (6).

Medication adherence is a key preventive measure in managing hypertension. According to the WHO, adherence is a measure of how well a person’s behavior complies with prescribed medication, dietary, or lifestyle changes agreed upon by a healthcare professional. Therefore, healthcare providers can aid in improving patient adherence to medication through interventions such as training and advising patients about the risks of uncontrolled hypertension, assessing patient barriers and fears, empowering patients in self-care, providing positive feedback on behavioral and clinical progress, avoiding complex drug regimens, and evaluating possible side effects of medication, while also strengthening family support (7). Iran is a developing country, and due to rapid social and economic development, along with changes in eating habits, a sedentary lifestyle, increasing life expectancy, and an aging population, the prevalence of hypertension is relatively high (8). Studies conducted in Iran have often taken a quantitative approach and sometimes emphasized a single factor or a minor aspect when investigating the factors affecting medication adherence in these patients. There is no comprehensive study addressing this issue and paying greater attention to residents of small towns.

Patients with hypertension exhibit different characteristics and possess a wealth of experiences in the field of research. Many of these experiences are subjective and may not be fully captured by quantitative methods. Therefore, in-depth interviews can offer a solution to this problem. The present qualitative study aimed to explain the facilitators affecting treatment and medication adherence from the perspective of patients with hypertension, providing researchers with insights into patients’ experiences. This study’s results can help identify patients’ actual needs in adhering to a drug regimen and underscore the necessity for comprehensive support from family, the healthcare system, and society. Effective disease management plays a significant role in the treatment, prevention of complications, cost reduction, and, ultimately, the enhancement of patients’ quality of life. Therefore, this study aimed to explore the effective facilitators of medication adherence observed by patients with hypertension.

## 2 Methods

### 2.1 Design

This qualitative study was conducted using conventional content analysis, a qualitative research technique designed to interpret the meanings expressed in the data derived from participants’ experiences (9).

### 2.2 Sample and setting

The sampling method was purposeful, with samples selected to maximize variation in age, gender, education, and disease duration in southeastern Iran. The inclusion criteria required participants to be at least 30 years old, have no known mental illness, and possess the ability to communicate with the interviewer. The data collection was conducted from September 2022 to April 2023, with each interview lasting between 30 and 60 min, depending on the situation and the consent of the participants. Finally, 25 people were enrolled in the study. The general characteristics of participants in the study are presented in Table 1.

**Table 1:** Demographic characteristics of the study participants.

Number	Gender	Age	Education
1	Female	66	Illiterate
2	Female	47	Secondary
3	Female	39	Diploma
4	Female	66	Illiterate
5	Female	36	Bachelor
6	Male	35	Diploma
7	Male	67	Diploma
8	Female	58	Associate degree
9	Male	36	Diploma
10	Male	40	Diploma
11	Male	49	Secondary
12	Female	44	Master of science
13	Male	47	Secondary
14	Female	51	Secondary
15	Male	72	Secondary
16	Female	62	Diploma
17	Male	70	Diploma
18	Female	64	Secondary
19	Female	33	Secondary
20	Male	85	Elementary
21	Male	62	Associate degree
22	Female	50	Associate degree
23	Male	63	Bachelor
24	Female	54	Elementary
25	Male	64	Illiterate

### 2.3 Data collection procedure

A semi-structured in-depth interview was used to collect data. First, a few questions were asked to create a friendly atmosphere. Subsequently, the interview process was guided by specific questions aligned with the study objectives. Some questions included: “How would you describe the pressure you experience?” “Do you feel you can take care of yourself?” “What factors have helped you adhere to the correct medication regimen, and why?.” Based on the participants’ responses, exploratory questions such as “Can you explain more?” were asked to delve deeper into the interview. Before the interviews, the study purpose and the data confidentiality were explained to the participants, and their consent was obtained. Based on the inclusion criteria, the data collection continued gradually until data saturation was achieved. Saturation occurs when all levels of coding are completed and no new conceptual information is obtained, allowing for the emergence of categories.

### 2.4 Data analysis

The interviews were transcribed verbatim and used as the main data for the research. The recorded voice was listened to, and the text was reviewed several times to gain an overall understanding of the content. Subsequently, the text was divided into meaningful units, condensed, and coded. The codes were then categorized based on similarities and differences into subcategories from which the main categories were extracted. An example of the analysis process used in this study is shown in Table 2. Microsoft Word software was used for data analysis.

**Table 2:** Example of qualitative content analysis process.

Subcategories	Primary code	Condensation	Meaning units
Constructive role of family	Supportive family	Following severe symptoms, the individual’s encouragement of their family to take medication and reminding them about the consequences of the illness	The first time the cardiologist diagnosed me with hypertension and prescribed medication, I experienced dizziness and a severe headache. At the insistence of my husband and children, I took the medication, as they emphasized the importance of taking the pills to prevent a stroke. (Participant No. 18)
		Children reminding and prompting timely and regular medication use, both in person and over the phone	My family often confronts me, asking, “Why did not you take your medicine?” My children even call to ensure I have taken my medication, and their reminders prompt me to take them. (Participant No. 8)
	The influence of motherhood	The sense of maternal responsibility and concern for their children’s future serve as strong motivations for mothers to adhere to their medication regimen.	I feel that if anything were to happen to me, it would greatly upset my daughter. For the sake of my children, I make a conscious effort to take my pills every day. Their hope and reliance on me give me the strength to adhere to my medication regimen. (Participant No. 3)
		The arrival of a new baby and the nurturing instinct it evokes serve as motivations for the mother to adhere to her medication regimen consistently.	Following childbirth, my blood pressure remained high, and for the well-being of my infant, I had to continue taking my medication as my infant was breastfeeding. (Participant No. 5)

### 2.5 Trustworthiness

The data trustworthiness was performed according to the Guba and Lincoln criteria. To increase the study’s credibility, the results were shared with some participants to ensure the accuracy of the concepts discussed in their interviews; for dependability, the research steps, decisions made, and the researcher’s engagement in data collection and analysis were accurately documented and recorded, A comprehensive description of the research process and the researcher’s engagement were provided. Additionally, the selection of participants was carefully considered to increase transferability. Quotations were also presented, reflecting their statements. The researcher’s continuous follow-up, active engagement, and appropriate interaction with participants were integral to confirming the validity of the data (10).

### 2.6 Ethical considerations

Participants were assured that their information would remain confidential and that they could discontinue the interview and refuse further collaboration if they wished. After obtaining written consent, individual interviews were conducted with the participants. The study protocol was approved by the ethics committee of Kerman University of Medical Sciences (Code of Ethics: IR. KMU. REC.1401.237).

## 3 Results

This study included 25 patients with hypertension from Mahan City, 52% of whom were female and 48% of whom were male. The patients’ education levels varied from illiterate to postgraduate degrees. The study identified effective facilitators for treatment and medication adherence, categorizing them into two main groups: individual and family factors and organizational support, as shown in Table 3.

**Table 3:** Theme, categories, and subcategories obtained from the results of content analysis.

Theme	Categories	Sub-categories
Facilitators of medication adherence	Individual and family factors	Intrinsic motivations
		Disease monitoring and tracking
		Constructive Role of Family
	Organizational support	The constructive role of government
		Constructive Role of Community

### 3.1 Individual and family factors

The participants identified internal motivations, such as fear of the consequences of the disease, symptom prevention, acceptance of reality, and drug literacy, as individual factors affecting adherence to the drug regimen and the treatment process of the disease. The fear of the consequences of illness, and in some cases, the firsthand experience of these consequences by the participants, was repeatedly mentioned as an important factor.

“*As my wife suffered from a stroke as a result of hypertension and was temporarily paralyzed, I feared that I might experience the same situation.” (Participant No. 25).*

One of the most important internal motivations for medication adherence was the desire to avoid the intolerable pain that comes with not taking the medication.

“*The Severe back pain makes me take medication; I just want to get rid of it.” (Participant No. 22).*

Additionally, patients’ belief in the severity of the disease and its sudden consequences can lead to the acceptance of the disease and serve as a starting point for adhering to the prescribed drug regimen.

*“We have become accustomed to dealing with hypertension, and we must always take medicines.” (Participant No. 5).*

The participants’ experiences indicate that knowledge of and trust in the effectiveness of the drugs are one of the motivational factors for adhering to the drug regimen. Some have reported feeling better and experiencing a disappearance of symptoms after taking the drugs.

*“With the drug, my blood pressure is regulated, and it proves to be very effective; it starts working 1–2 h later after taking it.” (Participant No. 7).*

In addition to drug treatment, desirable lifestyle changes and ongoing disease treatment monitoring also impact disease management. Some participants have changed their lifestyles to control blood pressure after diagnosis.

*“We have vegetables in the garden fruit trees of the same size as us, and after my wife had a stroke, we switched to low-salt just-add-water meals. As a result, my blood pressure has decreased.” (Participant No. 25).*

Practices such as periodic blood pressure checkups at home, timely medication use, and regular visits to the specialist affect the treatment process and help reduce the complications of the disease.

*“I visit the specialist for a check-up every three months and take medications according to the doctor’s instructions.” (Participant No. 6).*

Considering the family friendliness and support culture, simple interventions such as reminding patients and encouraging them to take their medication on time play an important role in improving patients’ treatment process and medication adherence.

*“If my daughter was not present at home reminding me to take my medication, I might not be here today.” (Participant No. 4).*

The future of their children and family planning were cited as motivating factors for adhering to medication.

*“For the sake of my infant, I would set alarms to ensure I took my medication on time so as not to compromise my infant’s well-being.” (Participant No. 3).*

### 3.2 Organizational support

The participants’ experiences highlight the importance of organizational support in facilitating individuals’ motivation to adhere to medication and treatment regimens. This support includes the constructive role of the government and the social context. The government’s provision of health services to the community has a significant impact on adherence to medication regimens and treatment behaviors in patients with hypertension. Furthermore, participants emphasized the crucial role of physicians and other healthcare providers in promoting adherence to medication and disease treatment through the provision of correct information and justification to the patients. The training they received from these healthcare providers also influenced their decision to continue the process.

*“Every month when I visit the doctor’s office, the doctor would spend 20 min with every patient, and after the examination, she would show us a clip related to the disease.” (Participant No. 6).*

For many participants, access to inexpensive medication emerged as one of the most important facilitators of adherence to the drug regimen. The cost of medicines can negatively affect adherence.

“*Hypertensive drugs are widely available and not too expensive.” (Participant No. 17).*

The role of society in providing appropriate infrastructure for treatment can affect the course of chronic disease, as patients often have to manage them for years. Having health insurance was cited as particularly helpful.

“*Whenever the doctor prescribes medication, I make sure to take it, and most of the time, I use my health insurance when visiting the hospital.” (Participant No. 14).*

Furthermore, the participants emphasized the significant role of the media in informing patients and providing them with accurate information about the factors affecting the disease, thereby contributing to the treatment process.

“*I prefer radio programs because I can listen to them while working. If there is a segment about blood pressure, I listen and then act accordingly.” (Participant No. 8).*

## 4 Discussion

The results of this study revealed that proper treatment and medication adherence in patients with hypertension could be affected by several factors. These include individual factors (such as motivational fears, disease acceptance, drug literacy, healthy lifestyle, disease monitoring, and follow-up) and family factors, including family support. Organizational support (including government support to provide inexpensive medicines, comprehensive support from the healthcare team, access to health insurance, and media training) also contributed to patients’ adherence to hypertensive drugs.

Fear of the consequences of the disease emerged as one of the most important motivations for patients to adhere to the drug regimen. This is supported by the findings of Bhandari et al. in Nepal, who identified fear of disease-related complications as a major driver in initiating drug use and adherence to treatment in participants (11). However, a study in the Philippines obtained contrasting results, indicating that disease-related consequences in patients’ relatives and families did not change patients’ medication adherence (12). Furthermore, disease acceptance was identified as an individual factor influencing the treatment of hypertension, a finding confirmed by a similar study in Tanzania (13).

Drug literacy and a positive attitude toward drugs were identified as the factors affecting the treatment of hypertension. Shen et al. in China supported these results, demonstrating that drug literacy was an independent predictor of medication adherence (14). These findings underscore the importance of patients’ role in adhering to treatment recommendations for hypertension, ultimately leading to disease control and a reduction in complications.

Adopting a healthy lifestyle was found to be another factor affecting the treatment of the disease, as making lifestyle changes is the first step in blood pressure management. This is consistent with the findings of Win et al. (15). Additionally, self-care practices such as blood pressure monitoring and regular visits to the doctor were identified as other factors affecting the treatment of hypertension. This finding is consistent with the study by Natalie et al. (16), which states that a healthy lifestyle and self-care practices could improve patients’ quality of life.

Family support, including support from a patient’s spouse and other family members, was an effective treatment and medication adherence factor. A similar study in Uganda emphasized the importance of family support in providing reminders for drug use and counseling on the importance of medication adherence in managing hypertension (17). When family members have sufficient knowledge about the nature of the disease and the principles of patient care, they can effectively influence the patient’s behavior and strengthen self-care practices.

Moreover, the quality of the patient-physician relationship, the physician’s communication style, and patient-centered treatment decisions were all identified as factors affecting adherence to the drug regimen and treatment. Trust was highlighted as a crucial element in these interactions, particularly in healthcare, a finding consistent with the study by Poulter et al. (18). The availability and affordability of medication emerged as an important motivating factor for adherence to the drug regimen, which was supported by a study in the Philippines (12). Additionally, the support of insurance organizations in reducing medical expenses was found to be an important factor influencing the treatment process, which is consistent with the study by Kisigo et al. (13). Consequently, the support of health organizations by increased insurance coverage and reduced out-of-pocket payments play an important role in improving the treatment process and medication adherence in these patients. Furthermore, continuous education provided by media was shown to help change attitudes and increase awareness across all different populations, thereby improving medication adherence. A similar study in Namibia confirmed this finding (19).

### 4.1 Limitations

In qualitative research, participants may not share all of their experiences on certain topics, and factors beyond the researcher’s control may influence their responses despite the research team’s efforts to communicate effectively. Another limitation was that this study was conducted qualitatively with a limited number of individuals with hypertension, making it challenging to generalize the findings to all individuals with this condition.

## 5 Conclusion

This qualitative study helped researchers explore patients’ experiences and beliefs, shedding light on factors influencing treatment and medication adherence in patients with hypertension. A key strategy identified was promoting patient education because it has been shown to impact treatment adherence positively. Therefore, the relationship between the healthcare provider and the patient was one of the important factors in adherence to the drug regimen. Consequently, it is important for physicians, especially during diagnosis, and other healthcare personnel involved in ongoing treatment and care to train patients regarding medication adherence and the factors affecting treatment. Additionally, the media, as a main and widely accessible source of information, plays a significant role in advancing community health, and leveraging this resource effectively can improve adherence to medication and treatment. Local community institutions, such as health and medical centers, have the resources, expertise, and networks to effectively help people adhere to hypertension treatment through various interventions. These institutions provide medical treatment services and can refer patients to higher levels of care, ensuring more accessible access to essential medications. Moreover, cooperating with community-based organizations can help promote awareness and disseminate information about hypertension.

## Data Availability

The raw data supporting the conclusions of this article will be made available by the authors, without undue reservation.
